# Imaging and Pathological Features of Idiopathic Portal Hypertension and Differential Diagnosis from Liver Cirrhosis

**DOI:** 10.1038/s41598-020-59286-8

**Published:** 2020-02-12

**Authors:** Zhen-Long Zhao, Ying Wei, Tai-Ling Wang, Li-Li Peng, Yan Li, Ming-An Yu

**Affiliations:** 1 Department of Interventional Ultrasound, China-Japan Friendship Hospital, Beijing, China; 2 Department of Pathology, China-Japan Friendship Hospital, Beijing, China

**Keywords:** Portal hypertension, Liver cirrhosis

## Abstract

Idiopathic portal hypertension (IPH) mimics liver cirrhosis in many aspects, and no efficient imaging method to differentiate the two diseases has been reported to date. In this study, the imaging and pathological characteristics were analysed for both IPH and cirrhosis. From January 2015 to March 2019, ultrasound, computed tomography (CT) and magnetic resonance imaging (MRI) images and pathological results from 16 IPH and 16 liver cirrhosis patients, as well as imaging results of 16 normal patients as a control group, were retrospectively reviewed. The age of the patients was 39 ± 20 years. There was a significant difference in the mean lumen diameter, wall thickness and ratio of thickness to diameter between the IPH and liver cirrhosis patients in the main and sagittal portal veins (P < 0.05), as well as in the lumen diameter and ratio of thickness to diameter between the IPH and liver cirrhosis patients in the Segment 3 (S3) portal vein (P < 0.05). In IPH patients, the main imaging changes were portal vein wall thickening, stenosis or occlusion, a low enhancement area along the portal vein in the delay phase in contrast-enhanced imaging, and a non-homogeneous change in T1WI. The corresponding pathological changes included interlobular vein thickening, stenosis, occlusion, portal area fibrosis, and atrophy or apoptosis of hepatocytes. The main imaging characteristic of liver cirrhosis was a nodular change in T1WI, and the related pathological change was pseudolobule formation. The imaging characteristics of IPH include thickening of the portal vein vascular wall, stenosis of the portal vein lumen and the absence of diffuse cirrhosis-like nodules. These imaging features have a definite pathological basis and could help make differential diagnoses between IPH and cirrhosis.

## Introduction

Idiopathic portal hypertension (IPH) is a relatively rare disorder characterized by portal hypertension without cirrhosis. The cause of IPH is complicated. The most common cause is certain kinds of drugs, such as thiopurine^1–3^ and arsenical salts^4,5^, and other causes include a series of autoimmune diseases, such as Hashimoto’s thyroiditis^6,7^, noninfectious hepatitis^7,8^ and rheumatoid arthritis^9^. Similar to cirrhosis, IPH can also lead to hepatic failure, splenomegaly, variceal bleeding and ascites^10^, making it difficult to differentiate IPH from cirrhosis. Liver biopsy has been the only way to diagnose IPH until now. A recent study showed that only a few special imaging examinations could possibly differentiate IPH from cirrhosis. The spleen/liver stiffness ratio measured by acoustic radiation force impulse (ARFI) elastography can differentiate between IPH and cirrhosis^11^. Delayed periportal enhancement on the contrast-enhanced ultrasound (CEUS) may be a characteristic of IPH^12^. The heterogeneous increase in arterial perfusion in the periphery of the liver on contrast-enhanced computed tomography (CT) imaging may be an important feature of idiopathic portal hypertension. The accumulation of Tc-99m galactosyl human serum albumin (GSA) was decreased in the periphery of the liver in single photon emission CT (SPECT) images, which may be one of the signs of IPH^13^. However, ARFI elastography lacks a unified parameter standard, and the other methods are associated with radiation and require a contrast agent. Because the treatment of IPH is completely different from that of cirrhosis, it is important to differentiate IPH from cirrhosis. Based on the respective characteristics of IPH and liver cirrhosis in regard to pathological changes, the imaging features may be different. In the present study, the imaging characteristics, including those of magnetic resonance imaging (MRI), CT and ultrasound, of IPH verified by pathology were retrospectively analysed and further compared with IPH pathology. The differential diagnosis between IPH and cirrhosis based on pathology and imaging was summarized.

## Results

The baseline characteristics of patients are summarized in Table 1. The average age of IPH patients was 39 ± 20 years, and 8 male patients and 8 female patients were included. Three patients underwent splenectomy. The underlying causes of IPH included autoimmune diseases such as psoriasis vulgaris (1 patient), the use of the Chinese herbal medicines Sedum aizoon (1 patient) and Stellera chamaejasme (1 patient), the use of oxaliplatin (2 patients), and eating snacks with food additives such as spicy strips (1 patient). No possible IPH-related causes were investigated in the other patients. Fifteen patients had splenomegaly. The average spleen pachydiameter was 5.4 cm ± 1.2 cm, and the length was 15.1 cm ± 3.2 cm. Two patients had high aspartate aminotransferase levels (41 U/L and 41.8 U/L). Three patients had low platelet counts (46 × 10^9^/L, 58 × 10^9^/L and 92 × 10^9^/L); MR examinations were performed in twelve patients, CT was performed in thirteen patients, and ultrasound was performed in all sixteen patients.

**Table 1:** Baseline Characteristics of Patients.

Parameter	IPH	Liver Cirrhosis	Healthy People
Age (y)^†^	39 ± 20	39 ± 20	39 ± 20
Sex (M/F)	8/8	8/8	8/8
Albumin (g/L)^†^	38.0 ± 4.1	35.5 ± 7.8	46.5 ± 7.2
Alanine aminotransferase (IU/L)^†^	20.9 ± 6.6	30.5 ± 17.6	15.1 ± 8.8
Aspartate aminotransferase (IU/L)^†^	27.6 ± 11.4	32.1 ± 15.5	19.5 ± 4.2
Platelet count (×10^9^/L)^†^	238 ± 239	282 ± 255	215 ± 64
Spleen pachydiameter (cm)	5.4 ± 1.2	5.2 ± 1.3	3.5 ± 0.4
Spleen length (cm)	15.1 ± 3.2	15.2 ± 3.3	11.2 ± 1.1

Note: IPH = idiopathic portal hypertension.

^†^Data at the time of each liver biopsy for the IPH group as well as the liver cirrhosis group and within 1 month for the healthy people group.

The clinical diagnoses of the 16 liver cirrhosis patients recruited as the cirrhosis group included alcoholic cirrhosis (4 patients), viral hepatitis-related cirrhosis (6 patients), primary biliary cirrhosis (4 patients) and congenital hepatic fibrosis (2 patients). Three patients underwent splenectomy. The Child-Pugh classification of the patients was class A for 14 patients and class B for 2 patients. The average spleen pachydiameter was 5.2 cm ± 1.3 cm, and the length was 15.2 cm ± 3.3 cm. There were no significant differences in spleen pachydiameter or length between the IPH group and the cirrhosis group. The detailed imaging evaluation of the liver and spleen is shown in Table 2.

**Table 2:** The imaging evaluation of the liver and spleen in the 3 groups.

Parameter	IPH	Liver Cirrhosis	Healthy People
Splenomegaly (pachydiameter >4.0 cm)	14/14 (100%) 2 cases of splenectomy	14/16 (87.5%)	0/16 (0%)
Portal-systemic collaterals	3/16 (18.8%)	4/16 (25%)	0/16 (0%)
Nodular surface	0/16 (0%)	10/16 (62.5%)	0/16 (0%)
FNH-like nodules	0/16 (0%)	6/16 (37.5%)	0/16 (0%)
Atrophy or hypertrophy of segments	7/16 (43.8%)	8/16 (50%)	0/16 (0%)
Portal vein enlargement (diameter ≥1.2 cm)	2/16 (12.5%)	3/16 (18.8%)	0/16 (0%)
Portal vein stenosis (diameter ≤ 0.6 cm)	2/16 (12.5%)	0/16 (0%)	0/16 (0%)
Hepatic artery enlargement (diameter >6.5 mm)	0/16 (0%)	2/16 (12.5%)	0/16 (0%)

### The imaging changes in the liver parenchyma

**Figure 1:**
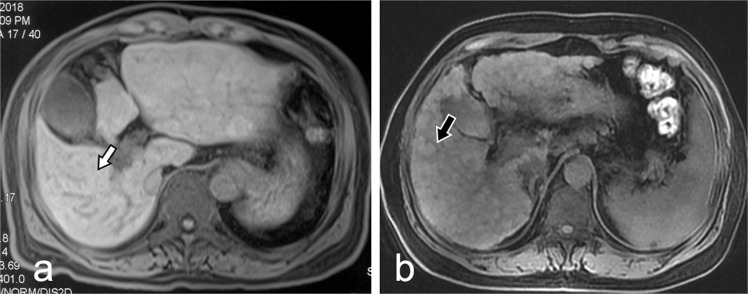
Image features of T1WI (TR 4.8, TE 2.4) in idiopathic portal hypertension (IPH) and liver cirrhosis patients. (**a**) Non-homogeneous change in the liver of a IPH patient (white arrow). (**b**) Nodular changes in the liver of a cirrhosis patient (black arrow).

Diffuse nodular changes on T1WI were shown in 63% (10/16) of the patients in the cirrhosis group but were not displayed in patients in either the healthy control group or the IPH group (Fig. 1). However, a non-homogeneous change was shown in the T1WI image in the IPH group, which was different from the homogeneous change in the healthy control group. No significant differences were found in T2WI or diffusion weighted imaging (DWI) images among the three groups.

### Imaging changes in the portal area

**Figure 2:**
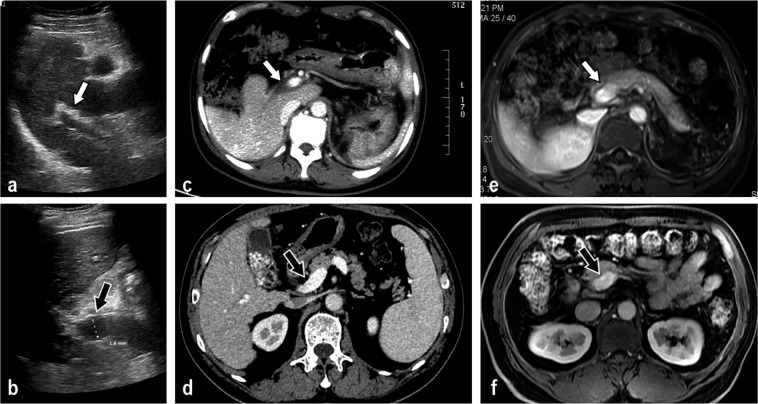
The change in main portal vein wall thickness in idiopathic portal hypertension (IPH) and cirrhosis patients based on ultrasound, CT and MR imaging (TR 4.8, TE 2.4). (**a**) The ultrasound image shows the thickened and rough vascular wall (white arrow) in an IPH patient. (**b**) The ultrasound image shows the thin vascular wall (black arrow) and dilated lumen of the main portal vein in a cirrhosis patient. (**c**) The contrast-enhanced CT image shows the thickened and rough lowly enhanced vascular wall (white arrow) in the delay phase in an IPH patient. (**d**) The contrast-enhanced CT image shows no significant change of the main portal vein vascular wall in the delay phase in a cirrhosis patient. (**e**) The contrast-enhanced MR T1WI image shows the thickened vascular wall in the delay phase (white arrow) in an IPH patient. (**f**) Contrast-enhanced MR image shows no significant change in the main portal vein vascular wall in the delay phase in a cirrhosis patient.

**Figure 3:**
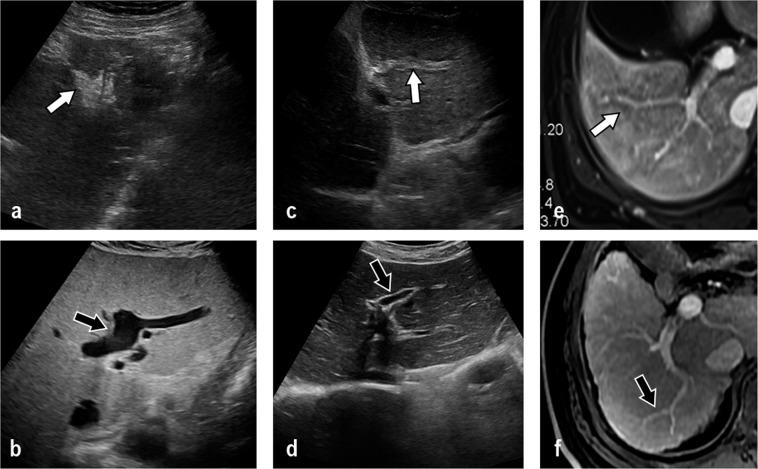
Images show the changes in the sagittal portion and other branches of the portal vein. (**a**) The ultrasound image shows the thickened vascular wall and nearly occluded lumen (white arrow) in the sagittal portion of the portal vein in an idiopathic portal hypertension (IPH) patient. (**b**) The ultrasound image shows the thin vascular wall (black arrow) and dilated lumen of the sagittal portion of the portal vein in a cirrhosis patient. (**c**) The ultrasound image shows the thickened vascular wall and nearly occluded lumen (white arrow) in the S3 portion of the portal vein in an IPH patient. (**d**) The ultrasound image shows the thin vascular wall (black arrow) and dilated lumen of the S3 portion of the portal vein in a cirrhosis patient. (**e**) The contrast-enhanced MR T1WI (TR 4.8, TE 2.4) image shows the thickened and rough vascular wall (white arrow) in an IPH patient. (**f**) The contrast-enhanced MR image shows no significant change in the S3 portal vein vascular wall in a cirrhosis patient.

The parameters of the main, sagittal and Segment 3 (S3) branches of the portal vein were compared among IPH patients, cirrhosis patients and healthy people. The lumen diameters of the main, sagittal and S3 branches of the portal vein in the IPH group and the healthy group were smaller than those in the cirrhosis group (P < 0.05). The vessel walls of the main and sagittal portal veins in the IPH group were thicker than those in the cirrhosis group (Figs. 2 and 3) and the healthy group (P < 0.05). The ratio of wall thickness to lumen diameter of the main and sagittal portal veins in the IPH group was greater than that in the cirrhosis group and the healthy group (P < 0.05). The detailed results are shown in Table 3.

**Table 3:** The means of lumen diameter, wall thickness and ratio of thickness to diameter in the 3 groups.

Part	Parameter	IPH	Liver Cirrhosis (vs IPH)	Healthy People (vs IPH)
Main portal vein	Diameter (mm)	9.71 ± 2.48	13.81 ± 2.68 (<0.001^*^)	9.69 ± 2.13 (0.999)
	Wall thickness (mm)	2.58 ± 1.15	1.39 ± 0.23 (0.002^*^)	1.66 ± 0.33 (0.018^**^)
	Ratio of thickness to diameter	0.29 ± 0.16	0.10 ± 0.02 (0.001^*^)	0.18 ± 0.36 (0.034^**^)
Sagittal portal vein	Diameter (mm)	7.29 ± 3.69	10.67 ± 2.16 (0.011^*^)	7.62 ± 0.94 (0.936)
	Wall thickness (mm)	3.41 ± 1.31	1.69 ± 0.34 ( < 0.001^*^)	2.01 ± 0.40 (0.002^**^)
	Ratio of thickness to diameter	0.59 ± 0.43	0.16 ± 0.03 (0.003^*^)	0.27 ± 0.06 (0.023^**^)
S3 portal vein	Diameter (mm)	2.67 ± 1.31	4.79 ± 1.46 (<0.001^*^)	2.41 ± 0.87 (0.785)
	Wall thickness (mm)	1.75 ± 0.72	1.55 ± 0.50 (0.635)	1.26 ± 0.26 (0.046^**^)
	Ratio of thickness to diameter	0.86 ± 0.60	0.34 ± 0.12 (0.010^*^)	0.56 ± 0.15 (0.163)

Note. IPH = idiopathic portal hypertension

The data are the means ± standard deviation.

^*^P < 0.05 IPH vs liver cirrhosis.

^**^P < 0.05 IPH vs healthy people.

A low enhancement area along the portal vein in the delay phase is shown in contrast-enhanced MRI (ceMRI) (Figs. 2e and 3e) and contrast-enhanced CT images (Fig. 2c) of IPH patients. This sign was not observed in the ceMRI (Figs. 2f and 3f) and CT (Fig. 2d) images of cirrhosis patients.

### Pathological changes in IPH and cirrhosis

**Figure 4:**
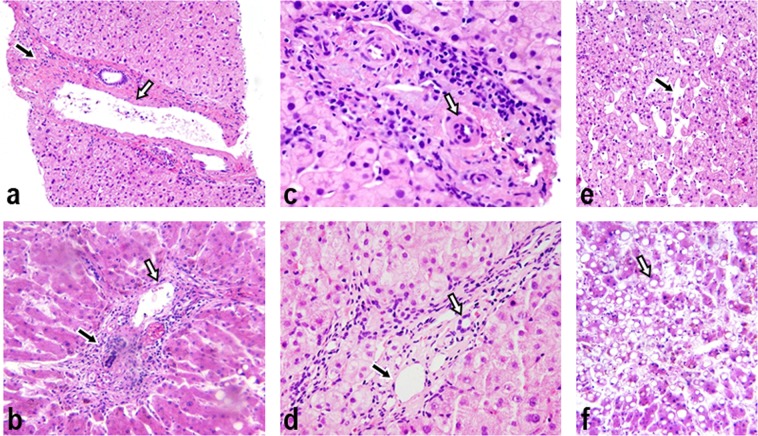
The pathological feature comparison between idiopathic portal hypertension (IPH) and cirrhosis. (**a**) Fibrosis in Glisson’s sheath (black arrow) and a thickened portal vein vascular wall (white arrow) are shown in an IPH patient (H&E, magnification ×200). **(b**) The lymphocytes aggregated in the portal area (black arrow), and the normal portal vein vascular wall (white arrow) of a cirrhosis patient is shown. No obvious fibrosis is shown in the portal area (H&E, magnification ×200). (**c**) The terminal portal venule is absent in the portal area in an IPH patient. The interlobular artery (black arrow) is shown (H&E, magnification ×400). (**d**) Portal area inflammation is shown in a cirrhosis patient, where the interlobular vein (black arrow) and interlobular artery (white arrow) are shown (H&E, magnification ×400). **(e**) Atrophy and apoptosis of some liver cells (black arrow) are shown in an IPH patient (H&E, magnification ×200). (**f**) Necrosis, oedema and fatty degeneration (white arrow) of liver cells are shown in a cirrhosis patient (H&E, magnification ×200).

**Figure 5:**
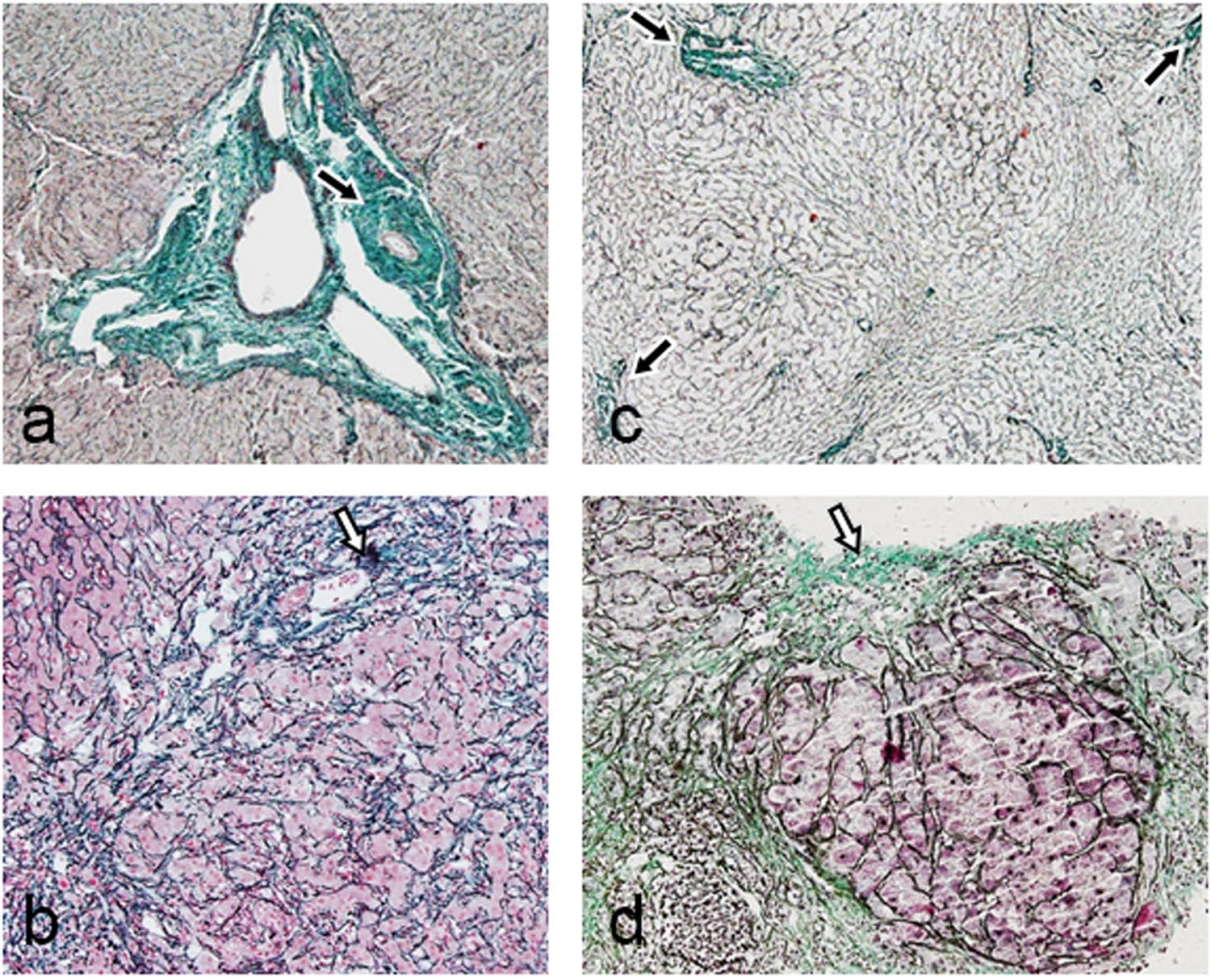
The pathological feature comparison between idiopathic portal hypertension (IPH) and cirrhosis. (**a**) Collagen fibres in the portal area in an IPH patient (black arrow) (Masson’s trichrome, magnification ×200). (**b**) The small amount of collagen fibres in the portal area and around the liver lobules in a cirrhosis patient (Masson’s trichrome, magnification ×200). (**c**) Collagen fibres in the portal area of an IPH patient (black arrow) (Masson’s trichrome, magnification ×40). **(d**) The collagen fibres around the pseudolobule are shown (white arrow) in a cirrhosis patient (Masson’s trichrome, magnification ×40).

Obvious fibrosis in the portal area was found in all pathological results of IPH patients (Fig. 4a), but this sign was absent in cirrhosis patients (Fig. 4b). An apparent increase in the amount of collagen fibres, particularly around blood vessels in the portal area, was shown in the livers of IPH patients (Fig. 5a), and the increase in collagen fibres was mostly distributed in the portal area (Fig. 5c). However, the collagen fibres were mostly distributed in the septa around the liver pseudolobules (Fig. 5b,d) beyond the portal area in cirrhosis patients. Interlobular vein occlusion was reported in 9 out of 16 IPH patients (Fig. 4c), dilation was reported in 10 patients, and wall thickening was reported in 6 patients. In contrast, most of the interlobular vein in cirrhosis patients showed no wall thickening or occlusion (Fig. 4d). In IPH cases, the liver cells showed atrophy or apoptosis (Fig. 4e) around the Glisson’s sheath, but necrosis was not found. In contrast, necrosis (75%), oedema and fatty degeneration (93.8%) of liver cells were observed in most of the cirrhosis patients (Fig. 4f). The results of pathological characteristics are shown in Table 4.

**Table 4:** The pathological results of liver biopsy of the two groups.

Parameter	IPH	Liver Cirrhosis	P value
Portal area fibrosis	16/16 (100%)	0/16 (0%)	<0.001*
Septal fibrosis	2/16 (12.5%)	15/16 (93.8%)	<0.001*
Interlobular vein occlusion	9/16 (56.3%)	2/16 (12.5%)	0.035*
Interlobular vein dilation	10/16 (62.5%)	8/16 (50%)	0.780
Interlobular vein thickening	6/16 (37.5%)	2/16 (12.5%)	0.239
Hepatocyte atrophy	15/16 (93.8%)	12/16 (75%)	0.381
Hepatocyte apoptosis	6/16 (37.5%)	3/16 (18.8%)	0.381
Hepatocyte necrosis	0/16 (0%)	12/16 (75%)	<0.001*
Hepatocyte oedema or fatty degeneration	2/16 (12.5%)	15/16 (93.8%)	<0.001*

Note. IPH = idiopathic portal hypertension.

^*^P < 0.05 IPH vs liver cirrhosis.

### Differences between IPH and liver cirrhosis based on imaging and pathology

In IPH patients, the main imaging changes were portal vein wall thickening, stenosis or occlusion, a low enhancement area along the portal vein in the delay phase in contrast-enhanced imaging, and a non-homogeneous change in T1WI imaging. The pathological changes included portal vein wall thickening, stenosis, occlusion, portal area fibrosis, and atrophy or apoptosis of hepatocytes.

The main imaging characteristic of liver cirrhosis was diffuse nodular change in T1WI MR imaging, and the related pathological change was pseudolobule formation. No imaging or pathological changes were shown in the portal vein in liver cirrhosis patients except for lumen dilation in some cases.

The specific findings related to portal hypertension in the IPH group included portal vein wall thickening and a low enhancement area along the portal vein in the delay phase ceMRI and contrast-enhanced CT. Other findings, such as portal vein lumen changes, splenomegaly and portal-systemic collaterals, were non-specific and could not differentiate IPH from liver cirrhosis.

## Discussion

Idiopathic portal hypertension (IPH) mimics liver cirrhosis in many aspects. The imaging examinations showed hepatic atrophy, splenomegaly and ascites in both diseases. The common clinical manifestations included abnormal liver-function tests, hypersplenism and varicosis^14^. Some patients presented anaemia and thrombocytopenia due to hypersplenism. To date, there are no effective imaging methods to differentiate between the two diseases. Therefore, IPH is easily misdiagnosed as liver cirrhosis. Liver biopsy is considered necessary to clarify the cause of portal hypertension^10^. However, liver biopsy is seldom performed on patients with liver cirrhosis and IPH because the patients often have hypersplenism and thrombocytopenia, and it is dangerous to perform the biopsy due to the high risk of haemorrhage. The treatment of these two diseases is different, and effective treatment requires a correct diagnosis. Therefore, it is important to find an efficient imaging method to differentiate between the two diseases. According to the results in the present study, there are obvious differences in both the parenchyma and portal area on imaging between IPH and liver cirrhosis. The diffuse nodular change in T1WI is a typical characteristic of liver cirrhosis. However, the imaging changes associated with IPH were mainly observed in the portal area. The imaging changes in IPH patients included wall thickening and an increased ratio of thickness to diameter in different sections of the portal vein, which were different from the imaging changes observed in cirrhosis patients and healthy people. The pathological results showed that portal vein wall thickening and obvious fibrosis in the portal area were common in IPH patients. In ultrasound, contrast-enhanced CT and MR images, the thickened vascular wall was rough and lowly enhanced, which is in accordance with the pathological changes. However, the vascular wall often remains smooth and is often too thin to be identified in CT and MR images in liver cirrhosis patients and healthy people.

Recent studies have shown that IPH is associated with many autoimmune diseases, such as systemic sclerosis^15^, systemic lupus erythaematosus^16^ and rheumatoid arthritis^9^. These results, combined with our own, indicate that IPH may be secondary to inflammation of connective tissue and vasculitis. The change in the portal area could be clearly demonstrated by pathological and imaging results in the present study. In addition, the low enhancement rough border around the portal vein in the delay phase in ceMRI and contrast-enhanced CT may be relevant to chronic inflammation and fibrosis of the portal area. As secondary changes, atrophy and apoptosis of liver cells induced by ischaemia were characteristic of IPH patients and ere displayed as heterogeneous changes in T1WI. Portal hypertension in IPH patients may result from fibrosis of the portal area and vessel wall thickening. Portal vein thrombosis was not found in our study by liver biopsy but has been reported in some studies^17,18^. The specific changes in IPH patients may not include portal vein thrombosis, and portal vein thrombosis may not be a specific sign of IPH. The changes in the portal vein vessel wall and its surrounding area caused by different factors may be the initiating factor and a specific characteristic of IPH.

The pathological basis of cirrhosis is the death of hepatocytes, extracellular matrix deposition, and vascular reorganization, in which hepatocytes and perisinusoidal stellate cells play the most important roles. The main pathological characteristic of liver cirrhosis is pseudolobule formation, which includes fibrous septa in the form of delicate bands or broad scars around lobules and parenchymal nodules encircled by fibrous bands. The above pathological changes could be in accordance with the nodular changes observed in T1WI images. However, there were no obvious changes in the portal area, as demonstrated by the pathological and imaging results in the present study. Portal hypertension in patients with cirrhosis results from increased resistance to portal flow at the level of the sinusoids and compression of central veins by perivenular fibrosis and expanded parenchymal nodules. Therefore, pseudolobule formation and nodular changes in T1WI are the main features of cirrhosis.

IPH has been a disease of undetermined aetiology until now. However, the main pathological and imaging changes concentrated on Glisson’s sheath in this study, so the main cause of IPH may be the damage of Glisson’s sheath and the portal venous system. Therefore, it is important that the interstitial part of the liver be focused on in both pathological examinations and imaging examinations.

In our experience, both ultrasound and MR can be used as good imaging methods to differentiate cirrhosis from IPH. Ultrasound has high spatial resolution and is convenient to observe the vascular wall and measure the diameter of the lumen. MR has high soft tissue resolution, and the thickened vascular wall can be observed clearly with a routine scan and contrast-enhanced T1WI. In addition, the non-homogeneous change and the absence of diffuse hepatic cirrhosis nodules in IPH patients can be clearly observed in the T1WI sequence.

Our research has certain limitations. The retrospective design creates a risk for selection bias. The absence of a validation control weakens the results of the study. The healthy people did not have pathological results for comparison. The number of cases was small, and more data on portal vein measurements are needed.

The imaging characteristics of idiopathic portal hypertension (IPH) include the absence of diffuse cirrhosis-like nodules, thickening of the portal vein vascular wall and stenosis of the portal vein lumen. These imaging features have a definite pathological basis and could help make differential diagnoses between IPH and cirrhosis. The interstitial changes in the liver should be concentrated on by radiologists.

## Methods

Our retrospective study was approved by the institutional review board of our hospital (China-Japan Friendship Hospital Institutional Review Board), and informed consent was waived by the institutional review board (China-Japan Friendship Hospital Institutional Review Board). The patients consented to the publishing of their anonymous examination results and radiological images. All methods were performed in accordance with the relevant guidelines and regulations.

### Patients

From January 2015 to March 2019, the ultrasound, CT and MR images as well as pathological results from 16 IPH patients were retrospectively reviewed. Idiopathic portal hypertension in the present study was characterized by portal hypertension; the absence of cirrhosis, as documented histologically in an appropriate liver specimen; and the absence of obstruction of the extrahepatic portal vein or hepatic venous outflow tract, sarcoidosis, schistosomiasis, congenital hepatic fibrosis, and other causes of cirrhosis. All patients had at least one clinical sign of portal hypertension (splenomegaly or hypersplenism, non-malignant ascites, minimally increased hepatic venous pressure gradient or portal-systemic collaterals). The patients did not have chronic liver diseases causing cirrhosis or non-cirrhotic portal hypertension, and they did not have congenital liver fibrosis, sarcoidosis or schistosomiasis, which cause non-cirrhotic portal hypertension^19^. The patients had patent portal and hepatic veins in Doppler ultrasound or CT scanning. The patients were diagnosed with IPH by pathological examination at China-Japan Friendship Hospital. Sixteen sex-matched liver cirrhosis patients with similar spleen pachydiameters (±0.5 cm) were recruited as the cirrhosis group for differential diagnosis. Sixteen age- and sex-matched healthy people were recruited as a healthy control group for imaging characteristics and liver parameters.

### Procedure of imaging and pathological examination

All IPH patients underwent contrast-enhanced ultrasound and MRI and/or CT. The ultrasound examination was performed in our department. The lumen diameter and wall thickness of the main, sagittal and S3 portions of the portal vein were measured in ultrasound images. Because the vessel wall is difficult to distinguish from the soft tissue around the vessel in liver, the wall thickness was measured as the thickest part of soft tissue around the vessel. S3 was chosen because it is horizontal relative to the ultrasound beam and is clearer than the vertical vessels. The pachydiameter and length of the spleen were measured with ultrasound. Three doctors experienced in ultrasound diagnosis performed the measurements, and the average of three measurements was used as the result. The doctors did not know the histological diagnosis of the patients during the measurement. The MR examinations were performed with a 1.5 T or 3 T scanner, and the sequence included T1WI (T1 weighted imaging), T2WI (T2 weighted imaging), fsT2WI (fat-saturated T2WI), DWI (diffusion weighted imaging) and contrast-enhanced T1WI (including artery phase, portal vein phase and delay phase). The contrast-enhanced CT included a routine scan, artery phase, portal vein phase and delay phase. The slice thickness of the CT and MRI was 3–5 mm. Three experienced radiologists reviewed the CT and MRI images and performed the measurements. Liver biopsy was performed in all IPH and liver cirrhosis patients. The histology of liver specimens was studied using H&E, Reticulin + Masson, D-PAS and cytokeratin 7 immunohistochemical staining. The “portal area fibrosis “ is reported if over 50% of the number of portal areas has collagen fibers deposition and the collagen fibers occupy over 50% of the area of them in Masson’s trichrome staining. Two doctors experienced in liver pathology made the pathological diagnosis.

The imaging characteristics, including liver parenchyma and portal area, of IPH patients were first evaluated and compared with those of patients in the cirrhosis group and healthy controls. Second, the imaging characteristics of IPH were compared with the pathological features of IPH. Third, a differential diagnosis was made between IPH and cirrhosis based on imaging and pathology.

### Statistical analysis

Statistical analyses were performed using SPSS version 24.0 (IBM, Armonk, NY, USA). The one-way ANOVA test (equal variances) and Welch’s ANOVA test (unequal variances) were used to test the differences between the means of continuous variables. Mann-Whitney U tests were used to test the differences of non-normal variables. P values < 0.05 were considered statistically significant.

### Consent to publish

The patients provided consent for the publishing of their anonymous examination results and radiological images.

## Data Availability

The datasets used and/or analysed during the current study are available from the corresponding author on reasonable request.
