# Author Correction: Imaging and Pathological Features of Idiopathic Portal Hypertension and Differential Diagnosis from Liver Cirrhosis

**DOI:** 10.1038/s41598-020-63868-x

**Published:** 2020-04-30

**Authors:** Zhen-Long Zhao, Ying Wei, Tai-Ling Wang, Li-Li Peng, Yan Li, Ming-An Yu

**Affiliations:** 1 Department of Interventional Ultrasound, China-Japan Friendship Hospital, Beijing, China; 2 Department of Pathology, China-Japan Friendship Hospital, Beijing, China

Correction to: *Scientific Reports* 10.1038/s41598-020-59286-8, published online 12 February 2020

This Article contains a typographical error in Table 2 in the Parameter column where,

Portal vein enlargement

(diameter ≥1.2 cm)

should read:

Portal vein enlargement

(diameter ≥1.5 cm)

